# Comparing growth velocity of HIV exposed and non-exposed infants: An observational study of infants enrolled in a randomized control trial in Zambia

**DOI:** 10.1371/journal.pone.0256443

**Published:** 2021-08-23

**Authors:** Obvious Nchimunya Chilyabanyama, Roma Chilengi, Natasha Makabilo Laban, Masuzyo Chirwa, Michelo Simunyandi, Luiza Miyanda Hatyoka, Innocent Ngaruye, Najeeha Talat Iqbal, Samuel Bosomprah

**Affiliations:** 1 African Centre of Excellence in Data Science (ACEDS), University of Rwanda, Kigali, Rwanda; 2 Research Division, Centre for Infectious Disease Research in Zambia, Lusaka, Zambia; 3 College of Science and Technology, University of Rwanda, Kigali, Rwanda; 4 Aga Khan University Hospital, Karachi, Pakistan; 5 Department of Biostatistics, School of Public Health, University of Ghana, Accra, Ghana; University of Cape Town, SOUTH AFRICA

## Abstract

**Background:**

Impaired growth among infants remains one of the leading nutrition problems globally. In this study, we aimed to compare the growth trajectory rate and evaluate growth trajectory characteristics among children, who are HIV exposed uninfected (HEU) and HIV unexposed uninfected (HUU), under two years in Zambia.

**Method:**

Our study used data from the ROVAS II study (PACTR201804003096919), an open-label randomized control trial of two verses three doses of live, attenuated, oral Rotarix^TM^ administered 6 &10 weeks or at 6 &10 weeks plus an additional dose at 9 months of age, conducted at George clinic in Lusaka, Zambia. Anthropometric measurements (height and weight) were collected on all scheduled and unscheduled visits. We defined linear growth velocity as the rate of change in height and estimated linear growth velocity as the first derivative of the mixed effect model with fractional polynomial transformations and, thereafter, used the second derivative test to determine the peak height and age at peak heigh.

**Results:**

We included 212 infants in this study with median age 6 (IQR: 6–6) weeks of age. Of these 97 (45.3%) were female, 35 (16.4%) were stunted, and 59 (27.6%) were exposed to HIV at baseline. Growth velocity was consistently below the 3^rd^ percentile of the WHO linear growth standard for HEU and HUU children. The peak height and age at peak height among HEU children were 74.7 cm (95% CI = 73.9–75.5) and 15.5 months (95% CI = 14.7–16.3) respectively and those for HUU were 73 cm (95% CI = 72.1–74.0) and 15.6 months (95% CI = 14.5–16.6) respectively.

**Conclusion:**

We found no difference in growth trajectories between infants who are HEU and HUU. However, the data suggests that poor linear growth is universal and profound in this cohort and may have already occurred in utero.

## Introduction

Impaired growth among infants remains one of the leading nutrition problems globally. In 2019, the prevalence of stunting among children under 5 years was estimated to be 21.3% (140 million children) globally and 33% (52 million children) in the sub-Saharan Africa region [1]. The global and sub-Saharan prevalence of stunting can be classified as high and very high, respectively [2]. The Zambia demographic health Survey (2014) (ZDHS) [3] reported a prevalence of 40% stunting among children under 5 and 56% for children between 18–23 months. Impaired growth is associated with an increased risk of mortality and morbidity among infants [4, 5]. Long-term and short-term effects of poor linear growth on the child included delay in motor skill development, impaired brain function, poor performance in school, and increased risk of morbidity and mortality [6, 7].

While several studies have shown a negative relationship between HIV infected children and attained growth [8–10], there were no studies, to the best of our knowledge, which studied the relationship between HIV exposure and linear growth velocity. There are concerns that children who are HIV exposed uninfected (HEU) are at risk of deficiencies in multiple micronutrients that play an important role in child growth and development [11]. Studies have shown that children who are HEU have an increased risk of mortality and morbidity than children who are HIV unexposed uninfected (HUU). This may be due to increased risk of prematurity and reduced care due to parental illness or death [12, 13]. Additionally, studies have also shown that children who are HEU have poor health outcomes compared to children who are HUU [14].

Characterizing growth trajectories among infants who are HEU is important in designing interventions aimed at improving the growth of these children. Previous studies investigating the relationship between impaired growth and HIV exposure have used attained growth for age, calculated as height for age (HAZ), but this has limitations. A HAZ measurement only reflects growth from birth to that particular point [15]. This may not give a proper indication of how the child has been growing. Growth velocity has been identified to be more robust and can identify growth problems earlier than attained growth for age [16–18].

In this study, we aimed to compare the rate of growth among children who are HEU and HUU under two years, enrolled in the ROVAS II clinical trial (PACTR201804003096919), using the World Health Organization (WHO) growth velocity standards (WHO 2009) [19]. We also evaluated growth trajectory characteristics by determining the peak height and age at which growth begins to falter.

## Materials and methods

### Study site and population

The University of Zambia Biomedical Research Ethics Committee, and the Zambian Ministry of Health approved the study. The study was conducted in accordance with the principles of the Declaration of Helsinki and in compliance with good clinical practice guidelines and is registered at ClinicalTrials.gov with registration number PACTR201804003096919. Written informed consent was obtained from all mothers of participating infants.

The ROVAS II study was conducted at George Clinic in Lusaka, between September 2018 and July 2021, a government health facility where the maternal child health (MCH) department, Antiretroviral Therapy (ART) clinic and the Centre for infectious disease research in Zambia (CIDRZ) research unit are co-located. The health centre catchment population is estimated to be around 145,230 [20]. The study site is a typical peri-urban setting in Lusaka. Mothers coming for their initial visit at the MCH between September and October 2018 were given information about the study. Motivated mothers were invited to the research unit, where informed consent was obtained. All pregnant women attending MCH were tested for HIV, and those who tested positives were immediately provided with life treatment regardless of their CD4 count in line with the WHO and national guidelines [21, 22]. Infants born from HIV positive mothers received the nationally recommended antiretroviral prophylaxis and were tested routinely at 6 weeks (enrolment), 6 months, 9 months and after cessation of breastfeeding or at two years of age. Infants who seroconverted for HIV were excluded from this analysis.

### Study design and participants

The ROVAS II is an open-label randomized control trial of two versus three doses of live, attenuated, oral Rotarix ^TM^ administered at 6 &10 weeks and at 6,10, plus 9 months of age. Infants were enrolled if; their parents consented to participate in the study and were eligible for Rotavirus vaccine immunization as per national immunization policy. Mothers were willing to have the child undergo study procedures such as full-course vaccination protocol anthropometric measurements and sample collection.

Anthropometrics measurements were collected according to the standard procedures by trained study nurses. Recumbent length was measured to the nearest 0.1 cm using a horizontal measuring board with a sliding foot piece. Infant’s weight and height were collected monthly from 6 weeks of age to 24 months as scheduled, but in addition to the scheduled visit, these measurements were collected each time the child was unwell and brought to the clinical research site.

### Statistical analysis

Summary statistics (mean and proportions) were used to describe participant’s baseline characteristics. This included infant age at the first visit, gender, birth weight, mother’s age at infant’s birth, maternal HIV status at infant’s birth, and HAZ. We calculated HAZ for each baseline height measurement available for each child using the WHO child growth standards WHO 2006 [23]. Stunting was defined as HAZ < − 2. Based on this cut-off, we calculated the proportion of children who were stunted at enrolment. We defined linear growth velocity as the rate of change in height. We included children who had at least three height measurements in the analysis.

We calculated growth velocity using two approaches. First, we calculated empirical growth velocity for each child as the difference between two successive height measurements divided by the corresponding age gap. We calculated the average 3-monthly age-specific growth velocity and 95% confidence interval (CI) using these values. The actual ages at which measurements were made were at times delayed (or advanced) compared to the target ages based on scheduled visits. This resulted in some measurement intervals being either longer or shorter than 91 days for a 3-month interval [24]. For this reason, we corrected the actual measurement age to target age using maximum tolerable difference. We adjusted standard errors for clustering of measurement within each child. The velocity and 95%CI for each age gap were presented using range plots for the entire sample and by sex and HIV exposure status.

Secondly, we estimated linear growth velocity as the first derivative of the mixed effect model with fractional polynomial transformations [25–27]. To account for the non-linear relationship between age and height, we applied the fractional polynomial model. This model has the form:

$$\beta_{0}+\sum_{j=1}^{m}\beta_{j}H_{j}(x)$$

Where $H_{1}(x)=x^{{(p}_{1})}$ and for j = 2,…, m,

$$H_{j}(x)=\left\{ \begin{array}{l} \;x^{p_{j}},\;p_{j}\neq p_{j-1}\\ H_{j-1}(x)ln(x),\;p_{j}=p_{j-1} \end{array}\right.$$

Where p_s_ are exponents selected from a sample space {-2, -1, -0.5, 0, 0.5, 1, 2, 3}.

The model assumes that *x* should be monotonically increasing, an assumption that holds true for infant height. We selected a parsimonious fractional polynomial model based on minimal deviance, according to a closed test procedure called the fractional polynomial selection procedure or function selection procedure as described in [25, 28]. This procedure is described further in Raysto (1990) [29].

After selecting the model, we fit the model in a mixed effect framework, and controlled for mother’s age, HIV exposure status, mothers’ education, mothers’ marital status, number of siblings, gestational age and gender to obtain predicted height for each child at each age. We then estimated the growth velocity as the numerical first derivative of the predicted height with respect to infant’s age. To characterize growth trajectory in terms of peak height and age at age growth begins to falter, we used the second derivative test. Infants who had less than three height measurements were excluded from the analysis due to the inability to calculate the rate of change of height over the duration of the study. The age and the height at that age were summarized to describe the trajectory characteristics. All analyses were performed using Stata 16.0 MP (Stata Corp, College Station, TX, USA).

## Results

### Characteristics of infants

The main study enrolled 214 infants aged between 5 and 11 weeks. We included 212 infants in this study after excluding 10 infants who were lost to follow up 2 who seroconverted for HIV during follow up. Hundred and sixteen (54.7%) infants enrolled were male while 96 (45.3%) where female, and 57 (26.9%) were HIV exposed. The median age of the mothers was 25 (IQR: 21–29). Hundred and twenty (56%) of the mothers had attained primary school education, while 39 (18%) and 53 (25%) had secondary or post-secondary education and no formal education, respectively. Most mothers, 174 (84%), were married, and only 34 (16%) were not married. Thirty-five (16.4%) infants were stunted (HAZ < -2) at enrolment, and there were no statistical differences in the level of stunting by mothers’ and infants’ demographic characteristics (Table 1).

**Table 1:** Infant nutritional status by background characteristics.

	n (%) of Total	n (%) Not Stunted	n (%) Stunted	p value
**Hiv Exposure**				
not exposed	155 (73.1)	132 (85.2)	23 (14.8)	0.171
exposed	57 (26.9)	44 (77.2)	13 (22.8)	
**Gender**				
Female	96(45.3)	85(88.5)	11 (11.5)	0.051
Male	116(54.7)	91(78.4)	25 (21.6)	
**Gestation**				
full term	198(93.4)	168(84.8)	30(15.2)	0.008
preterm	14(6.6)	8(57.1)	6(42.9)	
**Number of Siblings**				
1	61(28.8)	47(77)	14(23)	0.184
2–4	129(60.8)	112(86.8)	17(13.2)	
5 and above	22(10.4)	17(77.3)	5(22.7)	
**Mother’s age**				
<24	99(46.7)	81(81.8)	18(18.2)	0.268
24–35	100(47.2)	86(86)	14(14)	
36 and above	13(6.1)	9(69.2)	4(30.8)	
**Mother’s Education**				
No formal education	53(25)	43(81.1)	10(18.9)	0.521 1
Primary education	120(56.6)	98(81.7)	22(18.3)	
Secondary and post-secondary secondary	39(18.4)	35(89.7)	4(10.3)	
**Mother’s Marital**				
Single	35(16.5)	28(80)	7(20)	0.603
Married	177(83.5)	148(83.6)	29(16.4)	
**Total**	212(100)	176 (83)	36(17)	

### Growth velocity among HIV exposed and unexposed infants

**Fig 1:**
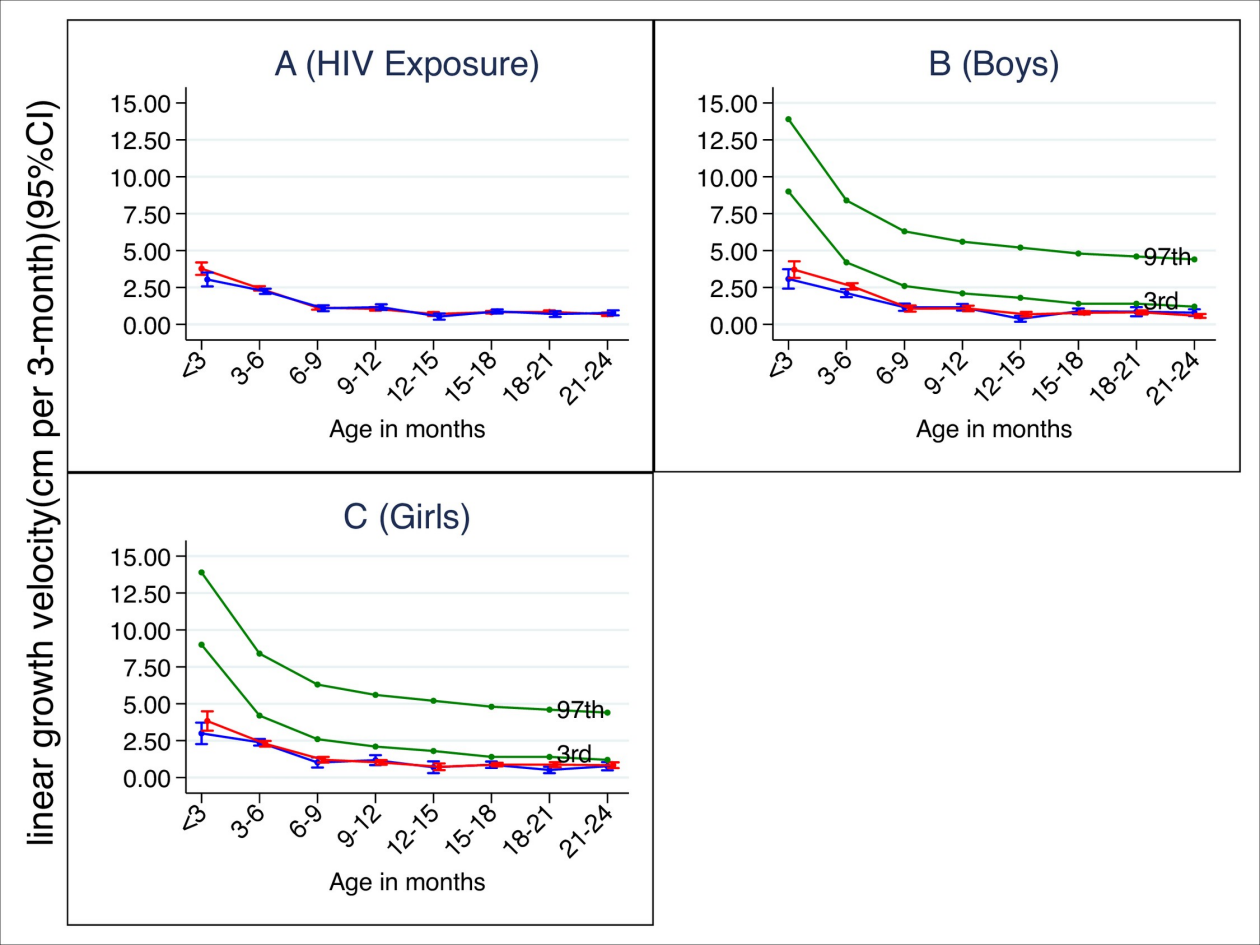
Three-monthly empirical linear growth velocity. In green are the WHO linear growth velocity standard (3rd and 97th percentiles), In blue is the three-monthly empirical growth of infants who are HIV unexposed and uninfected, while in red is the empirical growth of infants who are HIV exposed and uninfected with a 95% Confidence interval (CI). Overall growth velocity average per 3 months’ time by HIV exposure (a); Average growth velocity per 3 months for the boys (b); Average growth exposure for girls (c).

**Fig 2:**
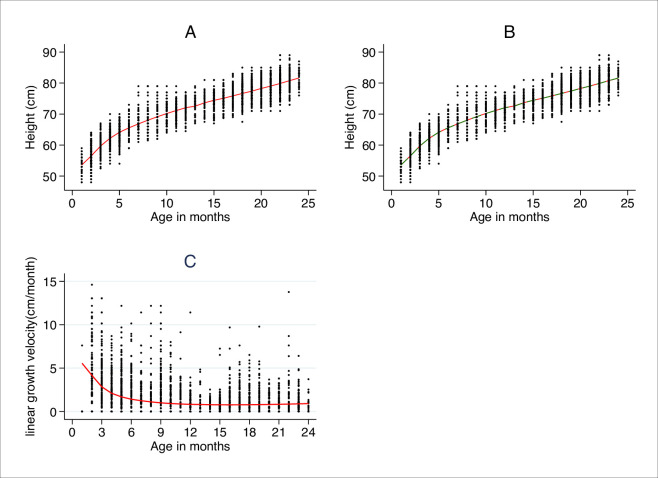
Observed and predicted linear growth mixed-effects model. Legend: in red and blue are average predicted growth curves for infants who are HUU and those who are HEU, respectively. Observed and predicted linear growth curves for all children (**a**); Observed and predicted mean linear growth for infants HUU and HEU (**b**). Observed and predicted linear growth velocity for infants who are HUU and HEU (**c**).

The 3 monthly average growth velocity is monotonically reducing over time. Between 0 and 6 months, the 3 monthly growth velocity is higher among infants who are HUU compared to those who are HEU. However, the difference is not statistically significant as all the 95% CI are overlapping and all p values based on a t test are non-significant (see Fig 2). Growth velocity was consistently below the 3^rd^ percentile of the WHO linear growth standard for boys who are HEU and HUU and for girls who are HEU and HUU see Fig 1, suggesting that growth retardation is universal in our cohort. There was no statistical difference in 3 monthly growth velocity between infants who are HEU and HUU at all time points as p values >0.05 see Table 2 below.

**Table 2:** Growth velocity for 3 months length increments.

	Total	Infants who are HUU	Infants who are HEU	p value ^1^
	GV cm/3 mo, (95% CI)	GV cm/3 mo, (95% CI)	GV cm/3 mo, (95% CI)	
0–3 months	3.48 (3.18–3.79)	3.65 (3.26–4.04)	3.08 (2.6–3.55)	0.11
3–6 months	2.4 (2.29–2.52)	2.45 (2.31–2.6)	2.28 (2.12–2.44)	0.14
6–9 months	1.11 (0.99–1.22)	1.11 (0.98–1.25)	1.09 (0.89–1.29)	0.90
9–12 months	1.08 (0.98–1.19)	1.05 (0.93–1.17)	1.17 (0.98–1.36)	0.24
12–15 months	0.65 (0.53–0.76)	0.7 (0.57–0.84)	0.53 (0.31–0.74)	0.16
15–18 months	0.84 (0.77–0.91)	0.83 (0.75–0.91)	0.88 (0.74–1.01)	0.84
18–21 months	0.79 (0.7–0.89)	0.84 (0.73–0.94)	0.7 (0.51–0.89)	0.29
21–24 months	0.72 (0.62–0.81)	0.69 (0.57–0.8)	0.78 (0.61–0.95)	0.42

^1^ T-test

Where GV: Growth velocity CI: Confidence Interval and mo: months

A fractional model of order p_1_ = 0 (log age) and p_2_ = 3 was selected against higher-order polynomials, using minimum deviance criteria. Thus, we fit a mixed effect model of the form;

$${height}_{ij}=\beta_{1}\;\log\left({age}_{ij}\right)+\beta_{2}\;{age}_{ij}^{3}+\beta_{3}hiv\;exposure+\beta_{4}\;{mother\;eductaion}_{i}+\beta_{5}\;{mother^{\prime }s\;age}_{i}+\beta_{6}\;mother^{\prime }s\;marital\;status+\beta_{7}\;{simblings}_{i}+u_{j}+\varepsilon_{ij}$$

Where *i* and *j* are visit and infants, respectively, the error terms from our model follow a gaussian distribution as can be seen in S1 Fig. There was no correlation between the predicted values residuals and the residual where normally distributed, as can be seen in S2 Fig. Further, visualization of individual growth shows that our model reflects both individual and population growth.

The estimated peak height and age at peak height were 73.8484 cm (95% CI = 14.91–16.13) and 14.6 months. This can be supported by the near-flat slight line after age month 144.5, as shown in Fig 2C. The predicted growth trajectory is between infants who are HUU and those who are HEU is similar as can be seen in Fig 2B. The height and age at peak height among infants who were HEU 14.74 months (95% CI = 14.35–5.14) and 73.16 cm (95% CI = 72.72–73.60) respectively and those for HUU were 14.48 months (95% CI = 14.12–14.84) and 74.16 cm (95% CI = 73.75–74.50) respectively. There is no statistical difference between the peak height and age at peak height between infants who are HEU and HUU, as can be supported by overlapping confidence intervals.

## Discussion

Our study showed no significant difference in the growth rate between infants who are HEU and HUU in our cohort, similar to the findings in Ross et al. (1995) [30]. Secondly, our study showed no association between baseline stunting and a child’s HIV exposure status in our cohort. Thirdly, we found that there is early linear growth faltering in infants both HEU and HUU. The growth velocity percentiles in the cohort were below the WHO 3^rd^ percentile, indicating poor growth among infants who are HEU and HUU [31], our finding is similar to the finding of Chilengi et al. (2019). In our cohort, growth rate falters around 15 months in both infants who are HEU and their HUU counterparts, slightly higher than 13.6 months as reported in a similar study [32].

The consistently lower growth velocity among infants in our cohort could be an indication of poor maternal nutrition [31]. It is suggestive that programmes aimed at averting poor growth among infants may should perhaps start during a mother’s pregnancy. Therefore, we advocate a need for studies that will focus on feeding practices among breastfeeding HIV infected mothers and HIV uninfected mothers. Several studies have suggested that an infant’s growth velocity may be affected by maternal nutrition status and duration of breastfeeding [33, 34]. That being the case, we are confident these findings would complement our findings.

Our study used fractional models because these models have been found to be more parsimonious compared to ordinary conventional polynomials [35]. Further, conventional polynomial models assume a smooth, monotonic curve between infants height and age. In contrast, other models such as splines or broken stick models assume a piecewise relationship between infants height and age, which is a biologically implausible relationship [36]. These models have been applied in longitudinal studies using the multilevel frames in studies on early growth trajectories [37] and BMI trajectories [38].

Our study has many strengths, such as repeated diverse anthropometric measures among comparable groups of infants who are HEU and HUU. To the best of our knowledge, this is a detailed description of growth trajectories among infants who are HEU and HUU in the same setting, in the duration of follow up, frequency of anthropometric assessment among infants. However, sample size limitations could result in low power and failure to identify true differences and associations. This study was conducted in George, a peri-urban area in Lusaka, and participants were not randomly selected. As a result, these results may not be generalizable to the rural populations. A post hoc sample size calculation based on [39, 40] indicates that our study achieved a power of 70% when using a Chi-squared test with 1 degree of freedom from a GEE analysis to determine whether the group slopes differ significantly at a significance level of 0.050 for 2 groups assuming each is measured 20 times. To achieve the power of 80,% the study needed to have enrolled 73 infants who are HUU and 145 infants who are HEU. Despite not collecting data on breastfeeding practices at each follow-up, all mothers in the study were breastfeeding at baseline and throughout the study as breast milk samples were collected at each visit. The national and WHO guidelines on HIV places no limit on the duration of breastfeeding among HIV positive women.

Since this study is observational, we note that there may be residual confounding social-economic factors, behavioural differences, or direct biological effect of HIV exposure on infant’s growth that ware not considered during the study’s design.

Despite demonstrating that infants who are HUU and HEU have similar growth patterns, the ROVAS II study was not initially designed to collect anthropometric data. The study did not collect data on how long an infant’s mother had been on anti-retroviral treatment (ART) or their viral suppression status. Duration on ART and viral suppression may be an important indicator of the mother’s quality of life, affecting the child’s growth.

## Conclusion

Our study showed no differences in the peak height velocity and age at peak height between HEU and HUU infants enrolled in the ROVAS II study. Further, we showed that the functional polynomial model of order 0 and 3 was the best fit model to describe linear growth among infants in our study. We also found a slower rate of growth and a high prevalence of stunting among Zambian infants. The data suggests that poor linear growth is universal and profound in this cohort and may have already been occurring before birth.

## Supporting information

S1 Fig Distribution of the error term of the mixed effect model. (TIF)

S2 Fig Correlation between residuals and the fitted values. (TIF)
