# Supplementary figures and images for: Comparing growth velocity of HIV exposed and non-exposed infants: An observational study of infants enrolled in a randomized control trial in Zambia
Source: PLoS One. 2021 Aug 23;16(8):e0256443. doi: 10.1371/journal.pone.0256443 (PMC8382174; doi:10.1371/journal.pone.0256443)

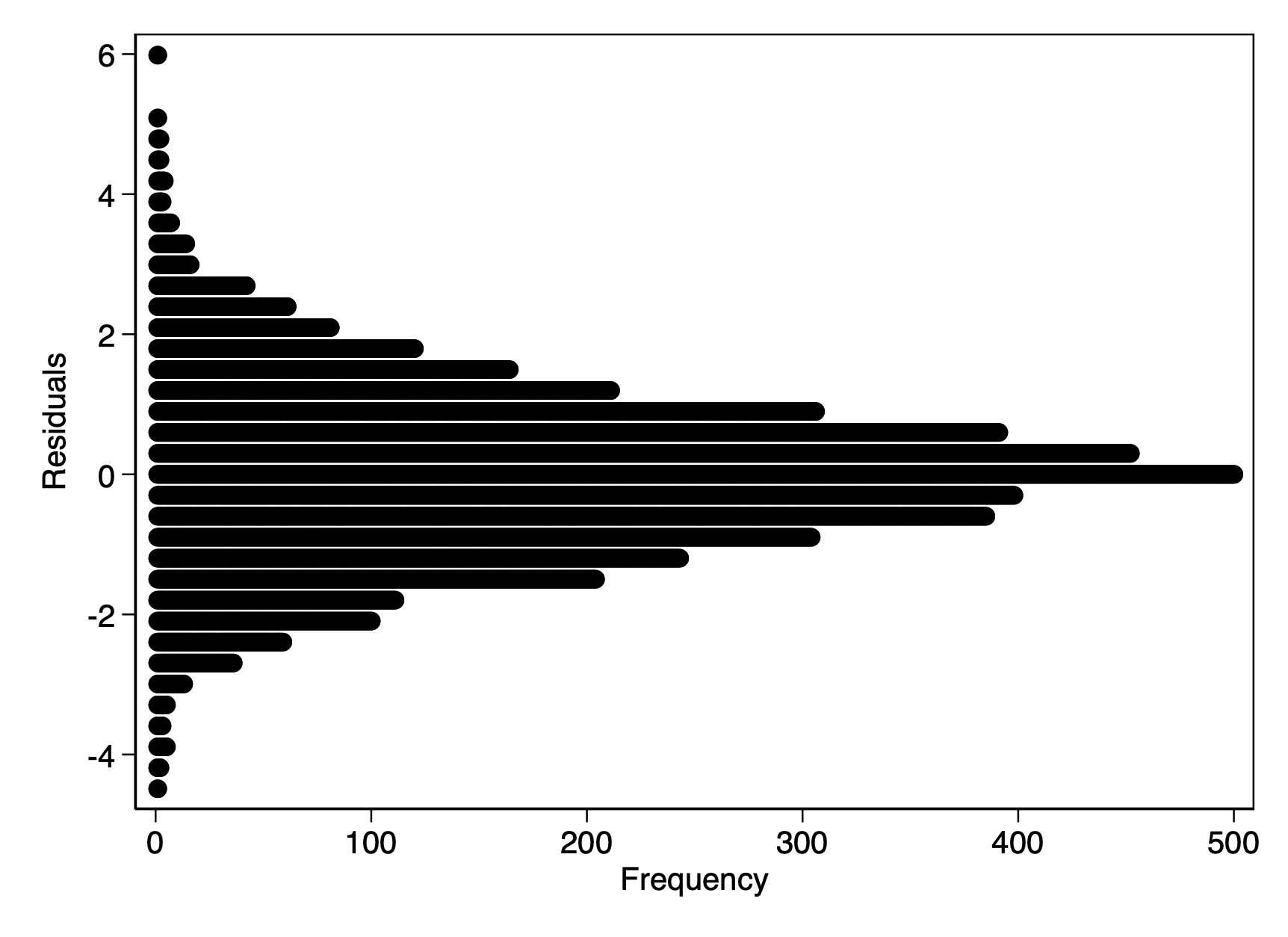

Supplement: S1 Fig — (TIF) [file pone.0256443.s001.tif]

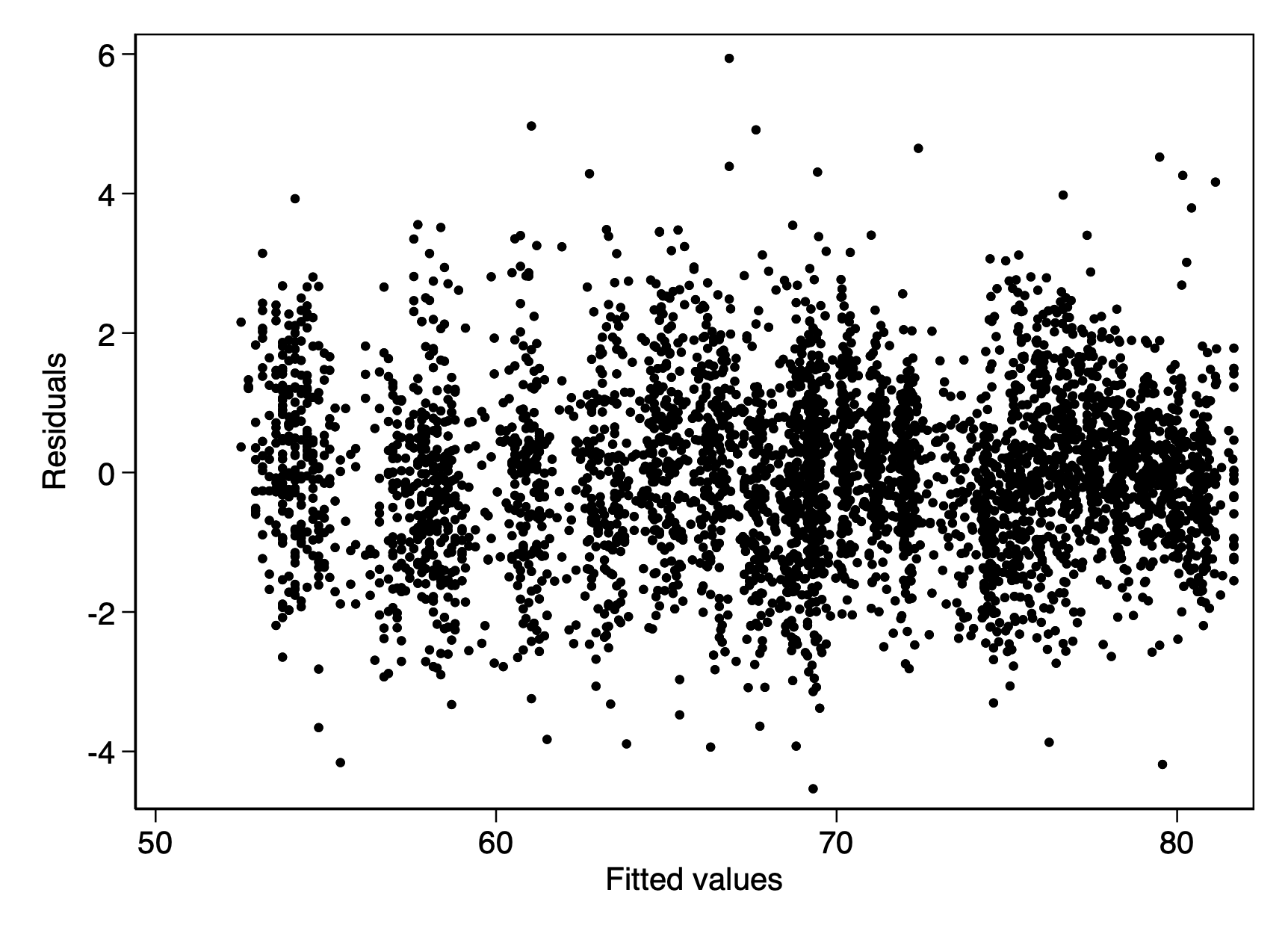

Supplement: S2 Fig — (TIF) [file pone.0256443.s002.tif]
